# Meta-Analysis of Randomized Controlled Trials Using Botulinum Toxin A at Different Dosages for Urinary Incontinence in Patients With Overactive Bladder

**DOI:** 10.3389/fphar.2019.01618

**Published:** 2020-01-15

**Authors:** Qin-Qin Gong, Yu-Qiong Xu, Jun Xu, Xiao-Yan Ding, Chong Guo

**Affiliations:** ^1^ Center for Women's Healthcare Sciences, Taihe Hospital, Hubei University of Medicine, Shiyan, China; ^2^ Department of Gynaecology, Peking University Shenzhen Hospital, Shenzhen, China; ^3^ Center for Gynaecology and Obstetrics, Taihe Hospital, Hubei University of Medicine, Shiyan, China

**Keywords:** urinary incontinence, neurogenic detrusor overactivity, idiopathic overactive bladder, Botulinum toxin A, meta-analysis

## Abstract

**Background:** Urinary incontinence (UI) is a common and refractory complication for patients with neurogenic detrusor overactivity (NDO) or idiopathic overactive bladder (IOAB).

**Objectives:** To evaluate the effect of Botulinum toxin A (BTX-A) based on different dosages strategy for UI.

**Method:** The MEDLINE, Ovid EMbase, The Cochrane Central Register of Controlled Trials (CENTRAL), China National Knowledge Internet (CNKI), and WanFang database were searched for relevant published randomized controlled trials (RCTs) between 1969 to September 31, 2018. All database were searched to identify relevant randomized controlled trials (RCTs) that investigated the clinical benefit of BTX-A for management of UI in patients with NDO and IOAB.

**Results:** This meta-analysis involved 19 original studies. The BTX-A was superior to placebo in reducing episodes of UI for NDO patients in all subgroups of different dosages for different durations, and also reduced maximum detrusor pressure in all kinds of 200U and 300U at 6 weeks. However, it increased post void residual in different dosages of 200U at 2 weeks. For IOAB patients, compared to placebo, BTX-A increased detrusor compliance for different dosages of 200U and 300U at 12 and 36 weeks, but it increased risk of urinary tract infections at other dosages.

**Conclusions:** This meta-analysis indicated that BTX-A 200U and 300U are more effective than placebo in the treatment of NDO, with minimal, local, and manageable adverse events. Furthermore, BTX-A 300U and 200U could also improve detrusor compliance of IOAB. However, more RCTs would still be necessary to explore the effect of BTX-A on management of UI in NDO and IOAB patients.

## Introduction

Overactive bladder (OAB) is defined as a series of symptoms (Abrams et al., 2002; White & Iglesia, 2016), including urinary urgency (usually accompanied by frequency and nocturia) and urinary incontinence (UI) in the absence of urinary tract infection (UTI) or other obvious pathology, according to the statement established by International Continence Society. The classification of OAB (Stewart et al., 2003; Lawrence et al., 2008) are generally considered two types: neurogenic detrusor overactivity (NDO) and idiopathic overactive bladder (IOAB). Almost 16.9% women suffer UI caused by OAB in the United States, which means that this disease has become a considerable common health issue with significant effects on women mentally and physically, of which mainly on account of urgency UI (Durden and Walker, 2018).

For management of UI in OAB patients, anticholinergic medicine is currently recommended as the first-line therapy (Garely and Burrows, 2002). Nevertheless, the anticholinergic medicine is increasingly inappropriate for long-term therapy of NDO and IOAB, which is reflected in the unsatisfied effect and potential complications such as vesicoureteral reflux and even renal failure (Majumdar, 2004; Asimakopoulos et al., 2012), and also the high socioeconomic cost is considerable. Therefore, meta-analyses (Cui et al., 2013; Mehta et al., 2013; Cui et al., 2015; Sun et al., 2015; Zhang et al., 2015; Zhou et al., 2015; Cheng et al., 2016; Gu et al., 2017) evaluating the therapeutic effect of botulinum toxin A (BTX-A) on UI in OAB patients have increased as well as the relevant RCTs in recent years, in which the BTX-A demonstrated a satisfied clinical benefit. (Flynn et al., 2004; Kessler et al., 2005; Kuo, 2005; Popat et al., 2005; Rajkumar et al., 2005; Schulte-Baukloh et al., 2005; Schmid et al., 2006) Furthermore, the BTX-A is recommended for management of UI in OAB patient by the American Urological Association (AUA) guidelines (Gormley et al., 2015), and the other interventions consist of education and behavior therapies. Nevertheless, the effect and safety of BTX-A is still controversial, furthermore, the clinical outcomes by different dosages also remains blank.

Therefore, we performed the systemic review and meta-analysis to evaluate the effect and safety of BTX-A at different dosages for the management of UI in patients with NDO and IOAB.

## Methods

This systemic review and meta-analysis was conducted in accordance with the Preferred Reporting Items for Systematic Reviews and Meta-Analyses guidelines (PRISMA) (Moher et al., 2009) and Cochrane Collaboration’s systematic review framework (Higgins JP, 2011).

## Search Strategy

The Ovid MEDLINE, Ovid EMbase, and the Cochrane Central Register of Controlled Trials (CENTRAL), China National Knowledge Internet (CNKI), and WanFang databases were searched between 1964 to September 31, 2018. All database were searched to identify relevant randomized controlled trials (RCTs) that investigated the clinical benefit of BTX-A for management of UI in patients with NDO and IOAB. All search strategy is described in **Supplementary Method 1**.

## Inclusion Criteria and Exclusion Criteria

RCTs were identified if the following criteria were met: (1) the patients with NDO or IOAB were confirmed; (2) patients >18 year; (3) studies compared BTX-A with placebo or BTX-A at different dosages, which reported in English and Chinese.

Studies were excluded for the following reasons: (1) stress incontinence; (2) duplicate studies; (3) for continuous outcomes, the standard deviations (SD) was still missing after contacting with the authors; (4) evaluated the clinical benefit of different injection sites only; (5) follow-up period was less than 1 week.

## Data Extraction

After independently reviewing the included studies by two reviewers (Hui-Yun Gu and Shuang Li), all following information were extracted: (1) first author, published year, regions, number of female patients, mean age, etiology of UI, and other basic disease; (2) the events, mean and SD of outcomes; (3) the effect outcomes and events in different time periods (2 weeks, 6 weeks, and 12 weeks) including UI episodes per week, maximum detrusor pressure (MDP), detrusor compliance (DC), and post void residual (PVR). The adverse events including urinary tract infections (UTI), urinary retention, hematuria, muscle weakness, and PVR-related catheterization were only recorded the events after follow-up without separately extracted according to different periods. The outcomes were expressed as two observation periods: short-term (≤12 weeks) and long-term (>12 weeks).

## Quality of Included Studies and Risk of Bias

For evaluating the risk of bias in RCTs, the Cochrane Collaboration’s tool (Higgins et al., 2011) was performed by two independent reviewers (Hui-Yun Gu and Shuang Li), which considers seven domains including adequacy of blinding of participants, sequence generation, allocation concealment, blinding of outcome assessment, selective outcome reporting, incomplete outcome data, and other potential sources of bias, in each item was graded as “high risk”, “low risk”, or “unclear”.

## Statistical Analysis

Dichotomous outcomes were expressed as the relative risk (RR) with 95% confidence interval (CI) (Deeks, 2002; Higgins JP, 2011), and continuous outcomes were expressed as mean difference (MD) (Higgins JP, 2011) with 95% CI. Both of them was bounded by P <0.05 for statistical significance. Forest plot was carried out to summarize the outcomes. Heterogeneity was tested using I2 tests (Melsen et al., 2014), in which the significance level was set to P< 0.1. I^2^ statistic provides an estimate of the percentage of inconsistency thought to be due to chance (Higgins JP, 2002). Initial analyses were performed using a fixed-effects model when I^2^< 40%, the random model was performed when I^2^> 40%.

The different dosages and types of IOAB and NDO were performed by subgroup analyses. All statistical analyses were performed using the Stata software (Versions, 12.0).

## Results

### Characteristics and Risk of Bias of Eligible Studies

**Figure 1:**
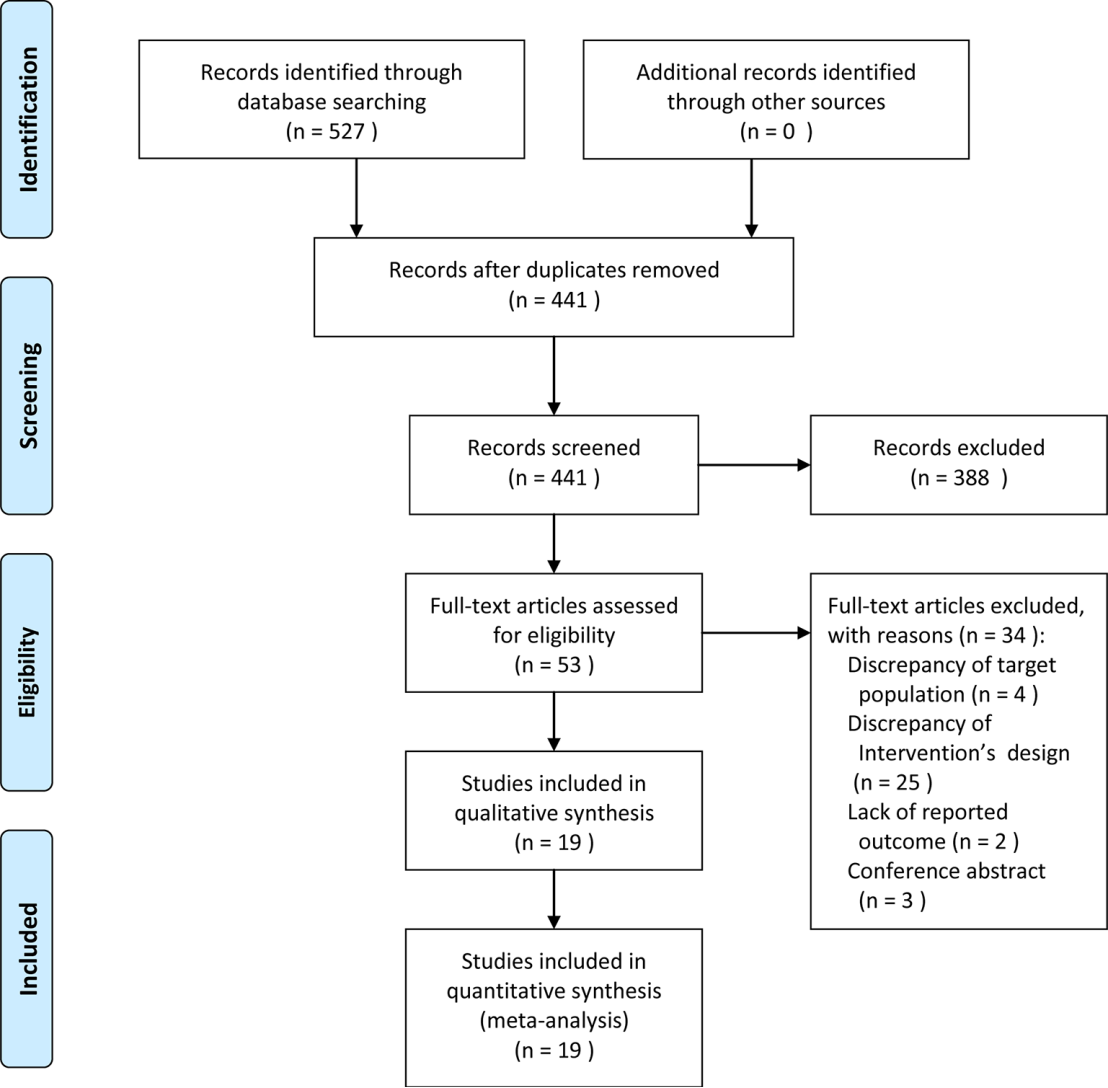
A flow diagram of the study selection process.

A total of 527 RCTs were initially identified, in which 86 studies were eliminated due to duplicates, then 441 studies were excluded in preliminary screening, therefore, only 53 articles were independently read in full text filtering. Ultimately, 34 studies were excluded (**Supplementary Method 2**), and a total of 19 articles (Schurch et al., 2005; Sahai et al., 2007; Brubaker et al., 2008; Cohen et al., 2009; Dmochowski et al., 2010; Altaweel et al., 2011; Cruz et al., 2011; Herschorn et al., 2011; Rovner et al., 2011; Denys et al., 2012; Ginsberg et al., 2012; Chapple et al., 2013; Ginsberg et al., 2013; Kennelly et al., 2013; Nitti et al., 2013; Rovner et al., 2013; Chen & Kuo, 2014; Sievert et al., 2014; Abdelwahab et al., 2015) were included in this meta-analysis (**Figure 1**).

Overall, 19 RCTs (Schurch et al., 2005; Sahai et al., 2007; Brubaker et al., 2008; Cohen et al., 2009; Dmochowski et al., 2010; Altaweel et al., 2011; Cruz et al., 2011; Herschorn et al., 2011; Rovner et al., 2011; Denys et al., 2012; Ginsberg et al., 2012; Chapple et al., 2013; Ginsberg et al., 2013; Kennelly et al., 2013; Nitti et al., 2013; Rovner et al., 2013; Chen & Kuo, 2014; Sievert et al., 2014; Abdelwahab et al., 2015) comprised 5,596 participants who were diagnosed as IOAB or NDO with UI were included in this study. Eight RCTs with 2,097 patients in NDO, and the remaining 11 RCTs comprised 3,499 IOAB patients were treated with one of these two interventions: BTX-A or placebo. The characteristics of included studies were presented in **Table 1**.

**Table 1:** Characteristics of individual study.

Study	Year	Region	No. of patients (female)	Ages Mean(SD)	Design	Classification of urinary incontinence	Basic diseases	Intervention	Follow-up (weeks)
Schurch	2005	Switzerland	59(23)	41	Randomized, doubled-blind	NDO	MS:6, SCI:53	Group 1: BTX-A 300U (19); Group 2: BTX-A 200U (19); Group 3: Placebo (21)	6
Sahai	2007	UK	34(19)	49.8, 50.8	Randomized, doubled-blind	IOAB	NA	Group 1: BTX-A 200U (16); Group 2: Placebo (18)	NA
Brubaker	2008	USA	43	64.7(14.5), 69.2(13.5)	Randomized, doubled-blind	IOAB	NA	Group 1: BTX-A 200U (28); Group 2: Placebo (15)	NA
Cohen	2009	USA	44	NA	Randomized, doubled-blind	IOAB	NA	Group 1: BTX-A 150U (22); Group 2: BTX-A 100U (22)	NA
Dmochowski	2010	USA	313(288)	58.8	Randomized, doubled-blind	IOAB	NA	Group 1: BTX-A 50U (56); Group 2: BTX-A 100U (55); Group 3: BTX-A 150U (50); Group 4: BTX-A 200U (52); Group 5: BTX-A 300U (55); Group 6: Placebo (43)	NA
Rovner	2011	USA	313(288)	58.8	Randomized, doubled-blind	IOAB	NA	Group 1: BTX-A 50U (57); Group 2: BTX-A 100U (54); Group 3: BTX-A 150U (49); Group 4: BTX-A 200U (53); Group 5: BTX-A 300U (56); Group 6: Placebo (44)	12, 36
Cruz	2011	Portugal	275(155)	46(13.1), 44.4(13.9), 46.9(13.4)	Randomized, doubled-blind	NDO	MS:154, SCI:121	Group 1: BTX-A 200U (92); Group 2: BTX-A 300U (91); Group 3: Placebo (92)	2, 6, 12
Herschorn	2011	Canada	57(23)	42.8	Randomized, doubled-blind	NDO	MS:19, SCI:38	Group 1: BTX-A 300U (28); Group 2: Placebo (29)	NA
Altaweel	2011	Saudi Arabia	22	NA	Randomized, doubled-blind	IOAB	NA	Group 1: BTX-A 200U (11); Group 2: BTX-A 100U (11)	NA
Denys	2012	France	199(87)	62.3, 61.7	Randomized, doubled-blind	IOAB	NA	Group 1: BTX-A 50U (23); Group 2: BTX-A 100U (23); Group 3: BTX-A 150U (30); Group 4: Placebo (31)	12
Ginsberg	2012	USA	416(245)	46(13)	Randomized, doubled-blind	NDO	MS:227, SCI:189	Group 1: BTX-A 200U (135); Group 2: BTX-A 300U (127); Group 3: Placebo (145)	6
Rovner	2013	USA	691(400)	45.9, 45.6, 46.2	Randomized, doubled-blind	NDO	MS:103, SCI:138	Group 1: BTX-A 200U (227); Group 2: BTX-A 300U (223); Group 3: Placebo (241)	6
Nitti	2013	USA	557(497)	61.7(12.7), 61(13.1)	Randomized, doubled-blind	IOAB	NA	Group 1: BTX-A 100U (278); Group 2: Placebo (272)	12
Kennelly	2013	USA	387(233)	46.4	Randomized, doubled-blind	NDO	SCI, MS	Group 1: BTX-A 300U (185); Group 2: BTX-A 200U (202)	6
Ginsberg	2013	USA	381(311)	49.7(12.1), 49.9(10.7), 50.2(10.7)	Randomized, doubled-blind	NDO	MS:381, SCI:310	Group 1: BTX-A 200U(227); Group 2: BTX-A 300U(223); Group 3: Placebo (241)	6
Chapple	2013	UK	548(473)	59.5(15.5), 59.2(14.1)	Randomized, doubled-blind	IOAB	NA	Group 1: BTX-A 100U (277); Group 2: Placebo (271)	NA
Sievert	2014	Germany	1105(970)	60.6(14.2), 60.1(13.6)	Randomized, doubled-blind	IOAB	NA	Group 1: BTX-A 100U (557); Group 2: Placebo (548)	NA
Chen	2014	Taiwan	72(29)	41.5	Randomized, doubled-blind	NDO	SCI	Group 1: BTX-A 300U (34); Group 2: BTX-A 200U (38)	NA
Abdelwahab	2015	Egypt	80(63)	31.35, 30.22	Randomized, doubled-blind	IOAB	NA	Group 1: BTX-A 100U (40); Group 2: BTX-A 200U (40)	12, 36

BTX-A, Botulinum toxin A; NDO, Neurogenic detrusor overactivity; IOAB, Idiopathic overactive bladder; MS, Multiple sclerosis; SCI, Spinal cord injury; NA, Not available.

The risk of bias in eligible studies was assessed by two independent authors (Hui-Yun Gu and Shuang Li) using the Cochrane Collaboration tool, and found that the 19 included RCTs were all graded as high-quality studies (**Table 2**).

**Table 2:** The risk of bias in the included studies.

Authors	Year	Random sequence generation	Allocation concealment	Blinding of participants and personnel	Blinding of outcome assessment	Incomplete outcome data	Selective reporting	Other bias
Schurch	2005	Low	Low	Low	Low	Low	Low	Unclear
Sahai	2007	Low	Low	Low	Low	Unclear	Low	Unclear
Brubaker	2008	Unclear	Unclear	Low	Unclear	Low	Low	Unclear
Cohen	2009	Low	Low	Low	Unclear	Low	Low	Unclear
Dmochowski	2010	Low	Unclear	Low	Low	Low	Low	Unclear
Rovner	2011	Low	Low	Unclear	Low	Low	Low	Unclear
Cruz	2011	Low	Low	Low	Low	Low	Low	Unclear
Herschorn	2011	Low	Low	Low	Low	Low	Low	Unclear
Altaweel	2011	Low	Low	Low	Low	Low	Low	Unclear
Denys	2012	Low	Unclear	Low	Low	Low	Low	Unclear
Ginsberg	2012	Low	Low	Low	Low	Low	Low	Unclear
Rovner	2013	Low	Low	Low	Low	Low	Low	Unclear
Nitti	2013	Low	Low	Unclear	Low	Low	Low	Unclear
Kennelly	2013	Low	Low	Low	Low	Low	Low	Unclear
Ginsberg	2013	Low	Low	Low	Low	Low	Low	Unclear
Chapple	2013	Low	Low	Low	Unclear	Low	Low	Unclear
Sievert	2014	Unclear	Low	Low	Unclear	Low	Low	Unclear
Chen	2014	Low	Low	Low	Unclear	Low	Low	Unclear
Abdelwahab	2015	Low	Unclear	Low	Unclear	Low	Low	Unclear

### Outcomes

All the results of following outcomes for NDO and IOAB patients were shown in **Table 3** and **Table 4**, respectively.

**Table 3:** Summary of results with different dosage at different observation points for NDO.

Outcomes	Number of RCTs	MD/RR	95%CI	I^2^ (%)	P for I^2^
**Episodes of Urinary Incontinence (UI) per Week**					
**2 weeks**					
BTX-A 200U VS. Placebo	1	-9.1	-14.10, -4.10	NA	NA
BTX-A 300U VS. Placebo	1	-6.1	-12.54, 0.34	NA	NA
BTX-A 300U VS. 200U	1	3	-3.30, 9.30	NA	NA
**6 weeks**					
BTX-A 200U VS. Placebo	3	-10.72	-13.40, -8.04	0	0.626
BTX-A 300U VS. Placebo	3	-11.42	-13.91, -8.93	50	0.135
BTX-A 300U VS. 200U	4	-0.38	-2.60, 1.84	0	0.765
**12 weeks**					
BTX-A 200U VS. Placebo	1	-8.5	-14.46, -2.54	NA	NA
BTX-A 300U VS. Placebo	1	-7.8	-13.73, -1.87	NA	NA
BTX-A 300U VS. 200U	1	0.7	-4.73, 6.13	NA	NA
**Maximum Detrusor Pressure (MDP)**					
**6 weeks**					
BTX-A 200U VS. Placebo	5	-33.01	-37.75, -28.27	0	0.998
BTX-A 300U VS. Placebo	5	-31.31	-35.79, -26.84	0	0.679
BTX-A 300U VS. 200U	6	1.16	-3.29, 5.60	0	0.831
**Detrusor compliance (DC)**					
**6 weeks**					
BTX-A 200U VS. Placebo	2	66.29	44.24, 88.34	0	0.837
BTX-A 300U VS. Placebo	2	56.51	35.00, 78.03	0	0.910
BTX-A 300U VS. 200U	2	-9.79	-35.00, 15.42	0	0.965
**Post void residual (PVR)**					
**2 weeks**					
BTX-A 200U VS. Placebo	2	93.87	63.91, 123.84	0	0.887
BTX-A 300U VS. Placebo	2	178.22	138.50, 217.95	0	0.751
BTX-A 300U VS. 200U	2	84.13	35.74, 132.51	0	0.727
Adverse Events*					
**Urinary tract infections (UTI)**					
BTX-A 200U VS. Placebo	5	1.44	1.27, 1.62	0	0.974
BTX-A 300U VS. Placebo	6	1.51	1.35, 1.70	0	0.720
BTX-A 300U VS. 200U	6	1.07	0.97, 1.18	0	0.909
**Urinary retention**					
BTX-A 200U VS. Placebo	4	5.85	3.84, 8.91	0	1.000
BTX-A 300U VS. Placebo	4	6.78	4.46, 10.30	0	0.872
BTX-A 300U VS. 200U	4	1.16	0.95, 1.43	0	0.438
**Muscle weakness**					
BTX-A 200U VS. Placebo	3	1.53	0.76, 3.06	69.9	0.036
BTX-A 300U VS. Placebo	4	3.01	1.50, 6.02	0	0.910
BTX-A 300U VS. 200U	3	1.75	1.03, 2.97	77.9	0.011
**Hematuria**					
BTX-A 200U VS. Placebo	5	1.59	0.97, 2.62	0	0.980
BTX-A 300U VS. Placebo	6	1.97	1.24, 3.13	0	0.984
BTX-A 300U VS. 200U	6	1.22	0.83, 1.80	0	0.902

NA, Not available.

**Table 4:** Summary of results with different dosage at different observation points for IOAB.

Outcomes	Number of RCTs	MD/RR	95%CI	I^2^ (%)	P for I^2^
**Maximum Detrusor Pressure (MDP)**					
**12 weeks**					
BTX-A 50U VS. Placebo	1	4.7	-3.49, 12.89	NA	NA
BTX-A 100U VS. Placebo	1	0.2	-7.55, 7.95	NA	NA
BTX-A 150U VS. Placebo	1	-4.2	-12.70, 4.30	NA	NA
BTX-A 200U VS. Placebo	1	5.7	-3.28, 14.68	NA	NA
BTX-A 300U VS. Placebo	1	0.1	-9.86, 10.06	NA	NA
BTX-A 100U VS. 50U	1	-4.5	-11.78,2.78	NA	NA
BTX-A 150U VS. 50U	1	-8.9	-16.98, -0.82	NA	NA
BTX-A 150U VS. 100U	1	-4.4	-12.03, 3.23	NA	NA
BTX-A 200U VS. 50U	1	1	-7.57,9.57	NA	NA
BTX-A 200U VS. 100U	1	5.5	-2.66, 13.66	NA	NA
BTX-A 200U VS. 150U	1	9.9	1.03, 18.77	NA	NA
BTX-A 300U VS. 50U	1	-4.6	-14.20, 5.00	NA	NA
BTX-A 300U VS. 100U	1	-0.1	-9.33, 9.13	NA	NA
BTX-A 300U VS. 150U	1	4.3	-5.57, 14.17	NA	NA
BTX-A 300U VS. 200U	1	-5.6	-15.88, 4.68	NA	NA
**36 weeks**					
BTX-A 50U VS. Placebo	1	2.6	-8.24, 13.44	NA	NA
BTX-A 100U VS. Placebo	1	8	-2.52, 18.52	NA	NA
BTX-A 150U VS. Placebo	1	3.4	-7.14, 13.94	NA	NA
BTX-A 200U VS. Placebo	1	3.8	-6.66, 14.26	NA	NA
BTX-A 300U VS. Placebo	1	8.1	-3.15, 19.35	NA	NA
BTX-A 100U VS. 50U	1	5.4	-1.62,12.42	NA	NA
BTX-A 150U VS. 50U	1	0.8	-6.25, 7.85	NA	NA
BTX-A 150U VS. 100U	1	-4.6	-11.15, 1.95	NA	NA
BTX-A 200U VS. 50U	1	1.2	-5.73,8.13	NA	NA
BTX-A 200U VS. 100U	1	-4.2	-10.62, 2.22	NA	NA
BTX-A 200U VS. 150U	1	0.4	-6.05, 6.85	NA	NA
BTX-A 300U VS. 50U	1	5.5	-2.58,13.58	NA	NA
BTX-A 300U VS. 100U	1	0.1	-7.55, 7.75	NA	NA
BTX-A 300U VS. 150U	1	4.7	-2.97, 12.37	NA	NA
BTX-A 300U VS. 200U	1	4.3	-3.26, 11.86	NA	NA
**Detrusor compliance (DC)**					
**12 weeks**					
BTX-A 50U VS. Placebo	1	65.5	23.24, 107.76	NA	NA
BTX-A 100U VS. Placebo	1	85.8	41.31, 130.29	NA	NA
BTX-A 150U VS. Placebo	1	36.4	-8.85, 81.65	NA	NA
BTX-A 200U VS. Placebo	1	104.5	47.52, 161.48	NA	NA
BTX-A 300U VS. Placebo	1	75.8	23.42, 128.18	NA	NA
BTX-A 100U VS. 50U	1	20.3	-28.92, 69.52	NA	NA
**Maximum Detrusor Pressure (MDP)**					
BTX-A 150U VS. 100U	1	-49.4	-101.21, 2.41	NA	NA
BTX-A 200U VS. 50U	1	39	-21.74, 99.74	NA	NA
BTX-A 200U VS. 100U	1	18.7	-43.62, 81.02	NA	NA
BTX-A 200U VS. 150U	1	68.1	5.24, 130.96	NA	NA
BTX-A 300U VS. 50U	1	10.3	-46.15, 66.75	NA	NA
BTX-A 300U VS. 100U	1	-10	-68.14, 48.14	NA	NA
BTX-A 300U VS. 150U	1	39.4	-19.32, 98.12	NA	NA
BTX-A 300U VS. 200U	1	-28.7	-96.87, 39.47	NA	NA
**36 weeks**					
BTX-A 50U VS. Placebo	1	60.3	16.08, 104.52	NA	NA
BTX-A 100U VS. Placebo	1	39.3	-6.18, 84.78	NA	NA
BTX-A 150U VS. Placebo	1	24.8	-30.63, 80.23	NA	NA
BTX-A 200U VS. Placebo	1	64.8	4.24, 125.36	NA	NA
BTX-A 300U VS. Placebo	1	60.8	11.64, 109.96	NA	NA
BTX-A 100U VS. 50U	1	-21	-62.98, 20.98	NA	NA
BTX-A 150U VS. 50U	1	-35.5	-88.10, 17.10	NA	NA
BTX-A 150U VS. 100U	1	-14.5	-68.16, 39.16	NA	NA
BTX-A 200U VS. 50U	1	4.5	-53.48, 62.48	NA	NA
BTX-A 200U VS. 100U	1	25.5	-33.45, 84.45	NA	NA
BTX-A 200U VS. 150U	1	40	-26.93, 106.93	NA	NA
BTX-A 300U VS. 50U	1	0.5	-45.43, 46.43	NA	NA
BTX-A 300U VS. 100U	1	21.5	-25.65, 68.65	NA	NA
BTX-A 300U VS. 150U	1	36	-20.81, 92.81	NA	NA
BTX-A 300U VS. 200U	1	-4	-65.83, 57.83	NA	NA
Adverse Events*					
**Urinary tract infections (UTI)**					
BTX-A 50U VS. Placebo	2	1.95	0.96, 3.98	0	0.675
BTX-A 100U VS. Placebo	5	2.55	2.09, 3.12	0	0.656
BTX-A 150U VS. Placebo	2	2.36	1.19, 4.68	0	0.355
BTX-A 200U VS. Placebo	2	2.68	1.46, 4.93	0	0.634
BTX-A 300U VS. Placebo	1	2.12	0.98, 4.58	NA	NA
BTX-A 100U VS. 50U	2	0.97	0.59, 1.59	18.5	0.268
BTX-A 150U VS. 50U	2	1.24	0.78, 1.98	0	0.594
BTX-A 150U VS. 100U	2	1.29	0.81, 2.05	0	0.443
BTX-A 200U VS. 50U	1	1.42	0.89, 2.25	NA	NA
BTX-A 200U VS. 100U	3	1.44	0.94, 2.20	0	0.685
BTX-A 200U VS. 150U	1	1.09	0.72, 1.67	NA	NA
BTX-A 300U VS. 50U	1	1.02	0.61, 1.71	NA	NA
BTX-A 300U VS. 100U	1	0.95	0.57, 1.57	NA	NA
BTX-A 300U VS. 150U	1	0.79	0.49, 1.27	NA	NA
BTX-A 300U VS. 200U	1	0.72	0.45, 1.14	NA	NA
**Urinary retention**					
BTX-A 50U VS. Placebo	1	3.84	0.47, 31.67	NA	NA
BTX-A 100U VS. Placebo	4	13.99	5.71, 34.30	0	0.946
BTX-A 150U VS. Placebo	1	12.04	1.65, 87.85	NA	NA
BTX-A 200U VS. Placebo	1	9.92	1.34, 73.29	NA	NA
BTX-A 300U VS. Placebo	1	10.95	1.50, 80.00	NA	NA
BTX-A 100U VS. 50U	1	2.04	0.74, 5.57	NA	NA
BTX-A 150U VS. 50U	1	3.14	1.22, 8.09	NA	NA
BTX-A 150U VS. 100U	1	1.54	0.75, 3.15	NA	NA
BTX-A 200U VS. 50U	1	2.58	0.98, 6.84	NA	NA
BTX-A 200U VS. 100U	2	1.34	0.66, 2.72	0	0.707
BTX-A 200U VS. 150U	1	0.82	0.42, 1.60	NA	NA
BTX-A 300U VS. 50U	1	2.85	1.10, 7.38	NA	NA
BTX-A 300U VS. 100U	1	1.4	0.68, 2.88	NA	NA
BTX-A 300U VS. 150U	1	0.91	0.48, 1.71	NA	NA
BTX-A 300U VS. 200U	1	1.1	0.56, 2.16	NA	NA
**PVR-related catheterization**					
BTX-A 50U VS. Placebo	2	1.06	0.26, 4.27	56.5	0.129
BTX-A 100U VS. Placebo	2	2.31	0.72, 7.37	58.2	0.122
BTX-A 150U VS. Placebo	2	2.4	0.79, 7.30	83.8	0.013
BTX-A 200U VS. Placebo	3	15.74	3.13, 79.31	0	0.985
BTX-A 300U VS. Placebo	1	14.93	0.89, 249.52	NA	NA
BTX-A 100U VS. 50U	2	2.28	0.73, 7.10	0	0.767
BTX-A 150U VS. 50U	2	2.89	0.98, 8.48	7.5	0.298
BTX-A 150U VS. 100U	3	1.22	0.57, 2.63	25.7	0.26
BTX-A 200U VS. 50U	1	3.95	1.17, 13.37	NA	NA
BTX-A 200U VS. 100U	1	1.94	0.77, 4.86	NA	NA
BTX-A 200U VS. 150U	1	1.06	0.49, 2.77	NA	NA
BTX-A 300U VS. 50U	1	3.05	0.87, 10.69	NA	NA
BTX-A 300U VS. 100U	1	1.5	0.57, 3.93	NA	NA
BTX-A 300U VS. 150U	1	0.82	0.36, 1.85	NA	NA
BTX-A 300U VS. 200U	1	0.77	0.35, 1.71	NA	NA
**Hematuria**					
BTX-A 100U VS. Placebo	2	1.38	0.78, 2.42	73.7	0.051

NA, Not available.

### Effectiveness

#### Episodes of Urinary Incontinence (UI) Per Week

##### For NDO in Short-Term Observation Period

**Figure 2:**
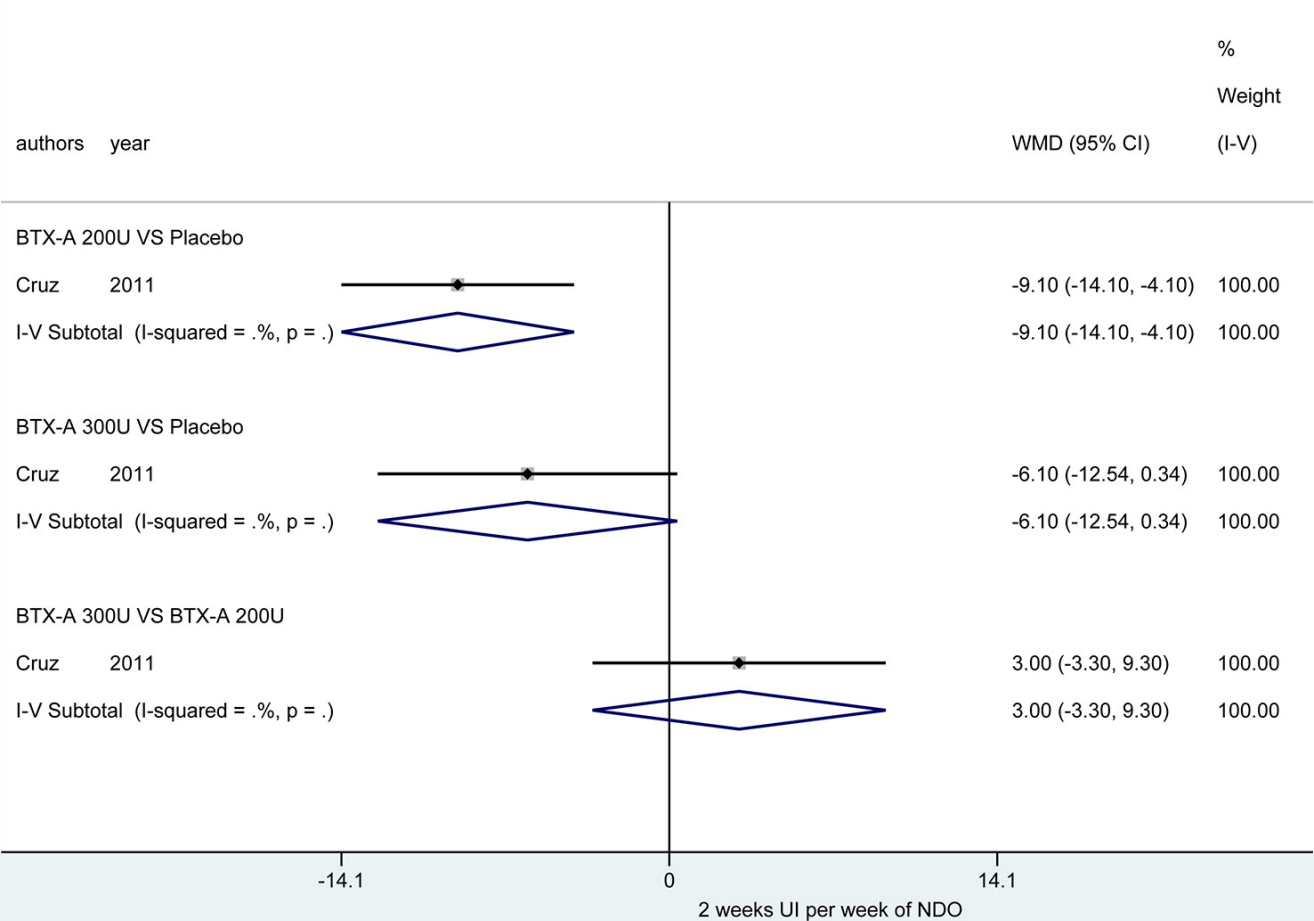
Forest plot of the changes of UI episodes per week of NDO at 2 weeks.

At 2 weeks. Only one study (Cruz et al., 2011) reported this outcome, and the BTX-A (200U) showed a significant improvement in reducing episodes of UI per week (MD = -9.10, 95% CI: -14.10, -4.10) compared to placebo. However, no significant effect was obtained in BTX-A (300U) compared to placebo (MD = -6.10, 95% CI: -12.54, 0.34). In addition, no significant effect between BTX-A at 300U and 200U was observed (MD = 3.00, 95% CI: -3.30, 9.30) (**Figure 2**).

**Figure 3:**
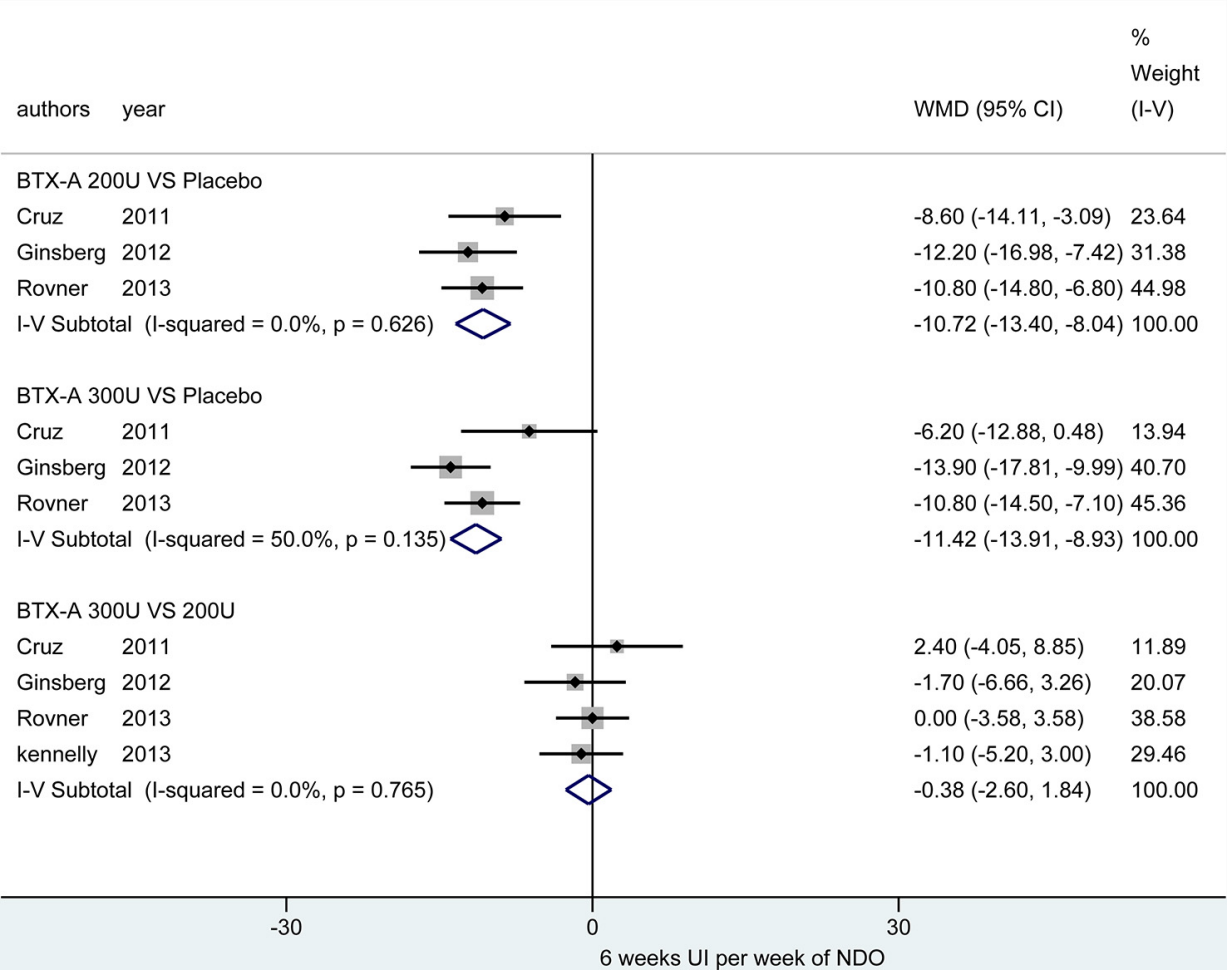
Forest plot of the changes of UI episodes per week of NDO at 6 weeks.

At 6 weeks. Four studies (Cruz et al., 2011; Ginsberg et al., 2012; Kennelly et al., 2013; Rovner et al., 2013) investigated these outcomes. **Figure 3** demonstrated that the BTX-A 300U (MD = -11.42, 95% CI: -13.91, -8.93) and BTX-A 200U (MD = -10.72, 95% CI: -13.40, -8.04) were significantly decreased the episodes of UI compared to placebo, nevertheless, the effect between BTX-A 300U and 200U (MD = -0.38, 95% CI: -2.60, 1.84) showed no significant reduction in episodes of UI (**Table 3**)

**Figure 4:**
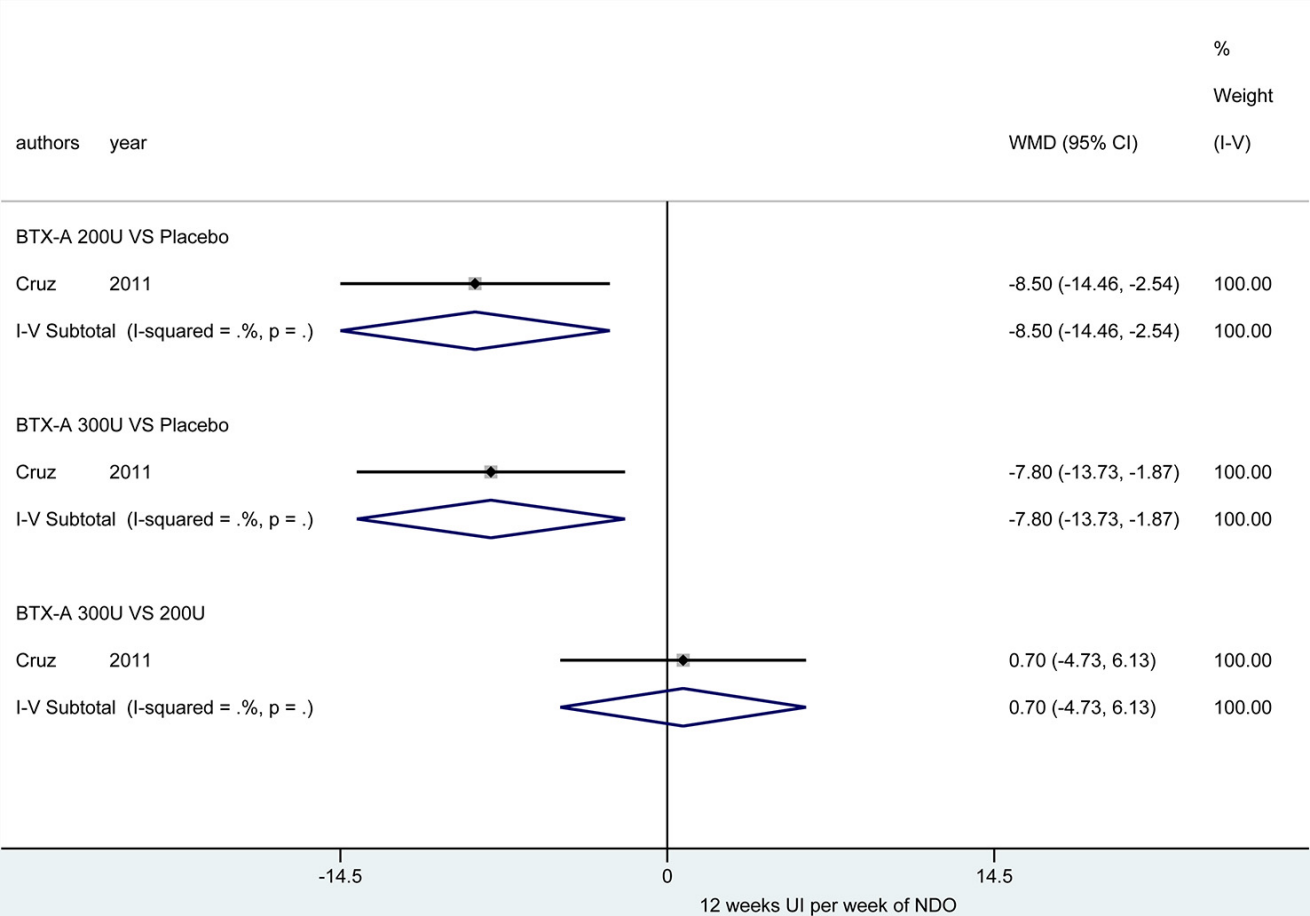
Forest plot of the changes of UI episodes per week of NDO at 12 weeks.

At 12 weeks. There was only one study (Cruz et al., 2011) reported this outcome. **Figure 4** showed that the effect from different dosages of BTX-A were significantly different compared to placebo (200U: MD = -8.50, 95% CI: -14.46, -2.54 and 300U: MD = -7.80, 95% CI: -13.73, -1.87), furthermore, no significant difference between 300U and 200U was observed (MD = 0.70, 95%CI: -4.73, 6.13) (**Table 3**).

#### Maximum Detrusor Pressure (MDP)

##### MDP for NDO in Short-Term Observation Period

**Figure 5:**
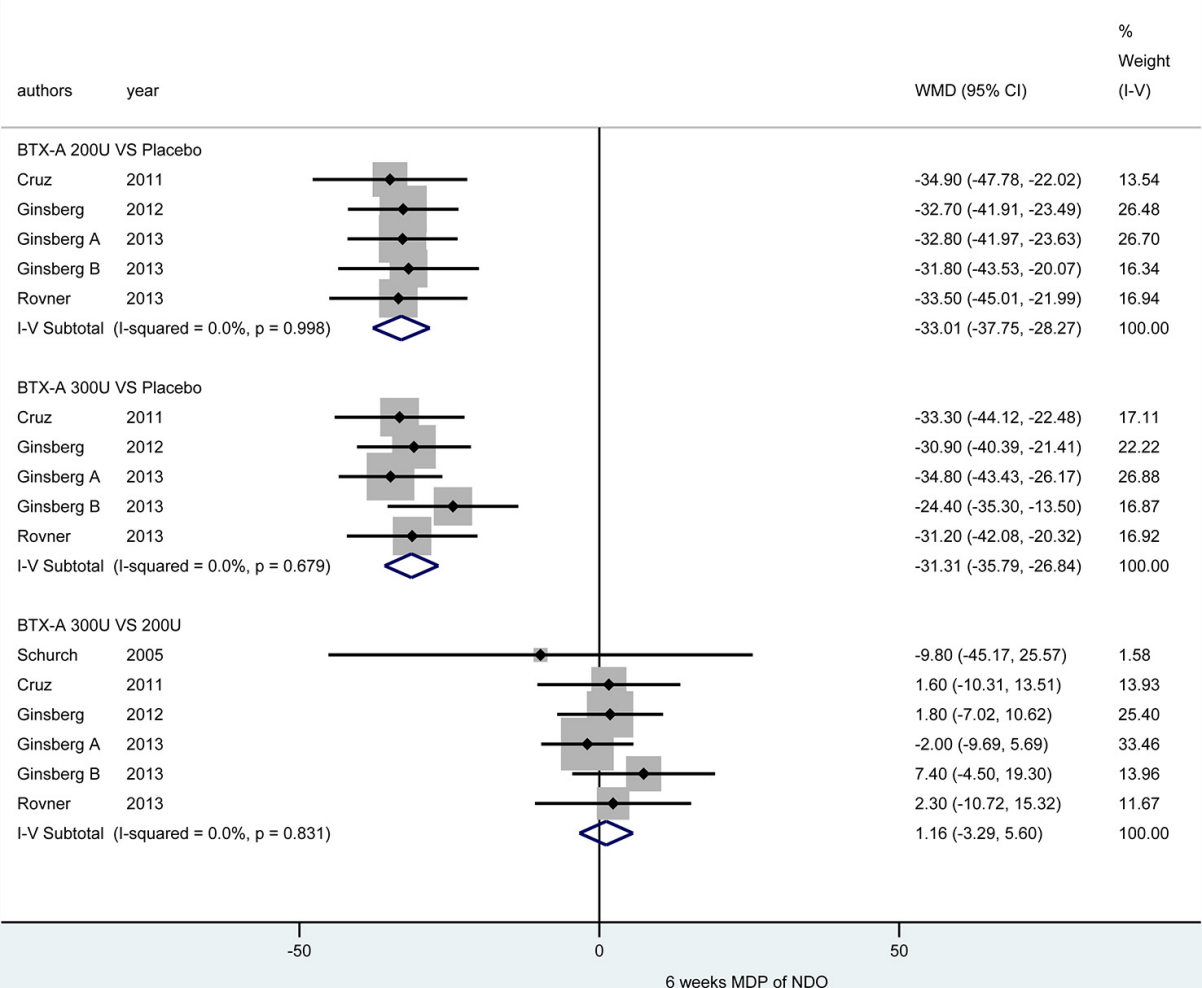
Forest plot of the changes of MDP of NDO at 6 weeks.

At 6 weeks. Five included studies (Schurch et al., 2005; Cruz et al., 2011; Ginsberg et al., 2012; Ginsberg et al., 2013; Rovner et al., 2013) reported this outcome. Compared with placebo, BTX-A at both 300U (MD = -31.31, 95% CI: -35.79, -26.84) and 200U (MD = -33.01, 95% CI: -37.75, -28.27) showed a significant effect in reducing MDP for NDO patients. However, the effect of different dosages from BTX-A was not significant (300U versus 200U: MD = 1.16, 95% CI: -3.29, 5.60) (**Figure 5**).

##### MDP for IOAB in Short-Term Observation Period

**Figure 6:**
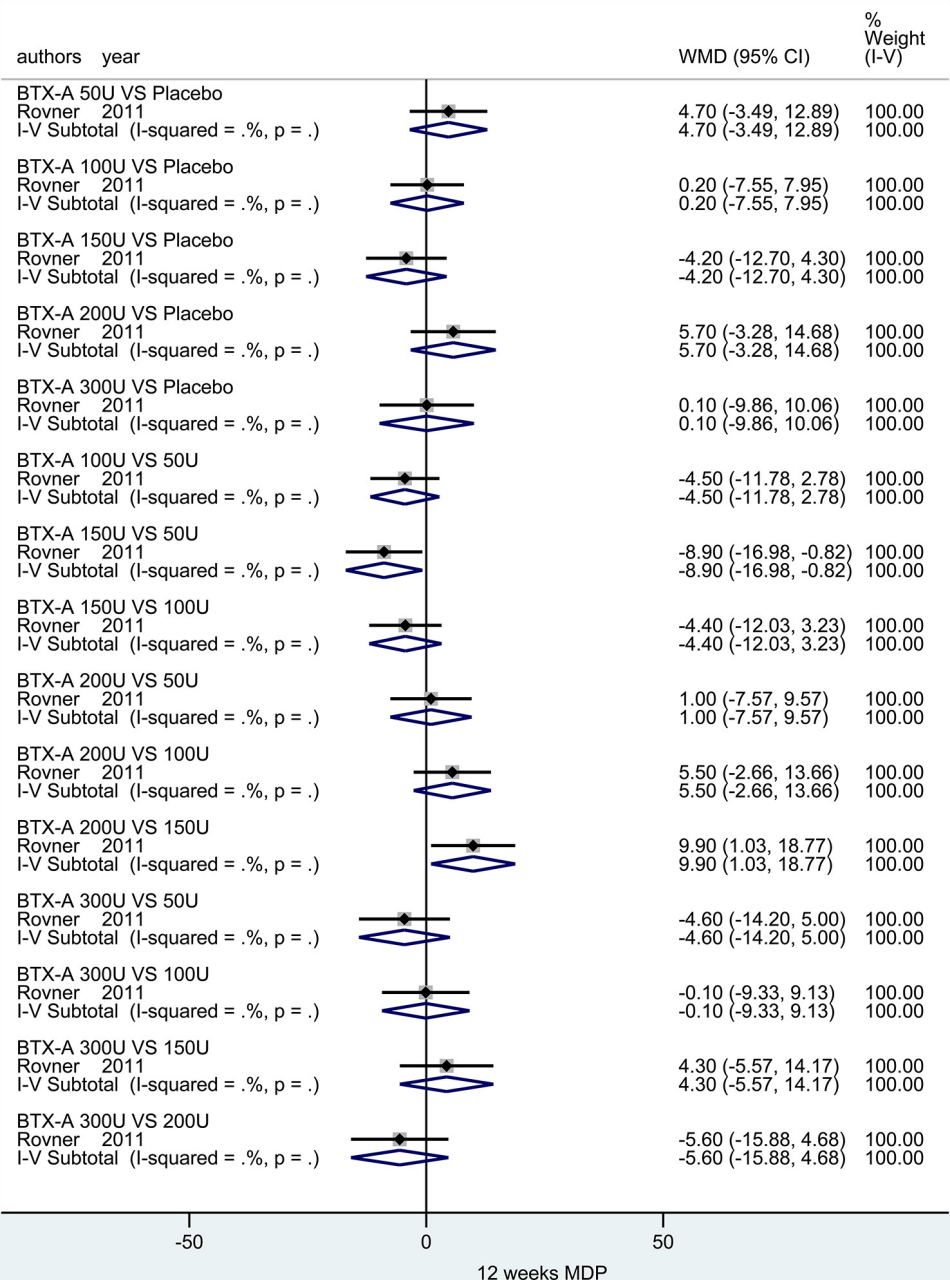
Forest plot of the changes of MDP of IOAB at 12 weeks.

At 6 weeks. There was only one study (Rovner et al., 2011) investigated this outcome. And no significant effect between different dosages (50U, 100U, 150U, 200U and 300U) was observed compared to placebo. In addition, statistic difference was merely obtained in two subgroups (BTX-A 150U vs. 50U: MD = -8.90, 95% CI: -16.98, -0.82 and BTX-A 200U vs. 150U: MD = 9.90, 95% CI: 1.03, 18.77), meanwhile, the other remaining results showed no statistical difference (**Figure 6**).

##### MDP for IOAB in Long-Term Observation Period

**Figure 7:**
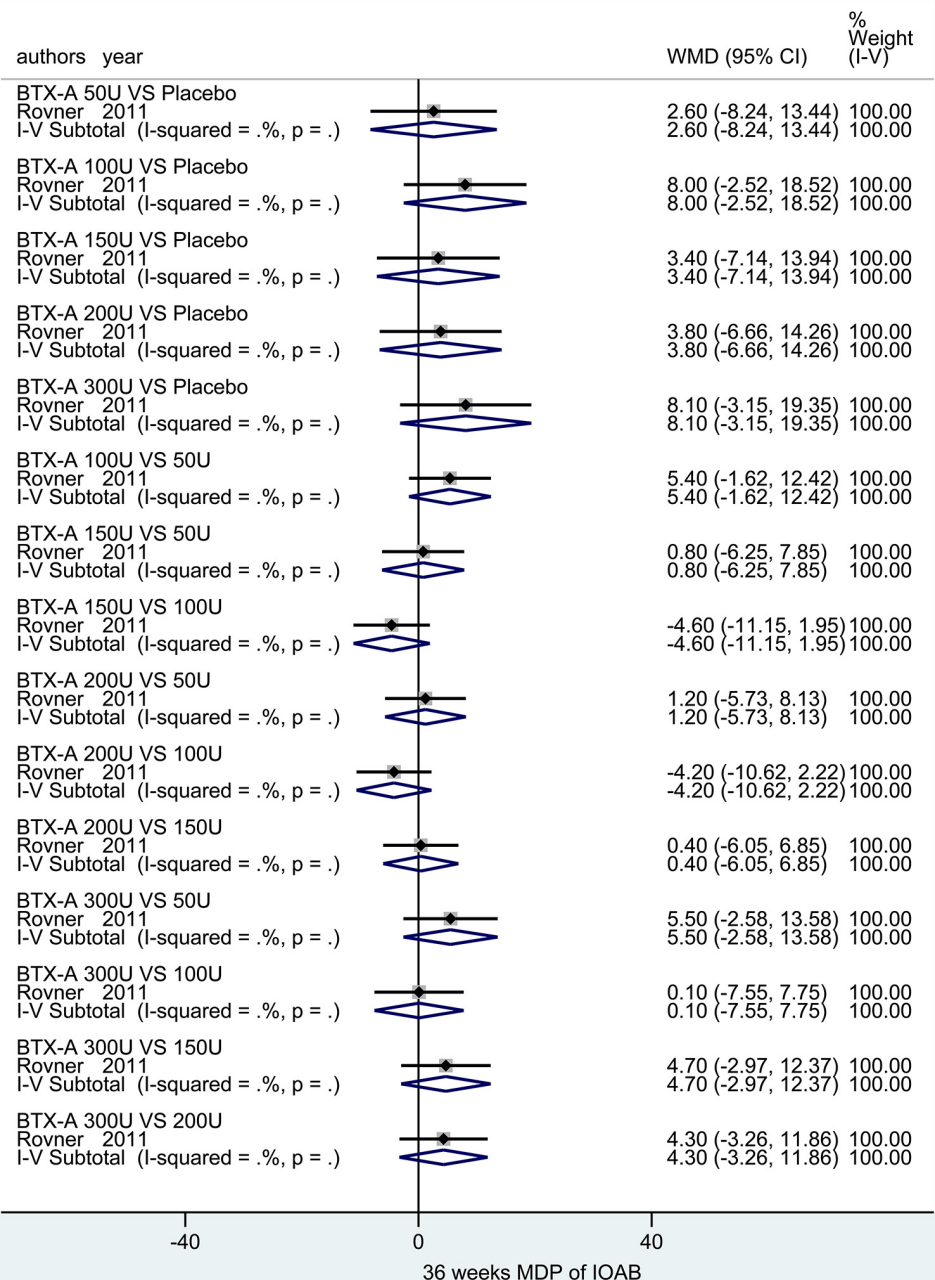
Forest plot of the changes of MDP of IOAB at 36 weeks.

At 36 weeks. Only one study (Rovner et al., 2011) reported the outcome. No significant difference was observed in neither different dosages at BTX-A (50U, 100U, 150U, 200U, and 300U) nor compared to placebo in IOAB patients (**Figure 7**).

##### PVR for NDO in Short-Term Observation Period

At 2 weeks. Two included studies (Cruz et al., 2011; Rovner et al., 2013) investigated the outcomes. **Table 3** showed that BTX-A (200U: MD = 93.87, 95% CI: 63.91, 123.84; and 300U: MD = 178.22, 95% CI: 138.50, 217.95) significantly increased PVR compared to placebo. Furthermore, significant difference was also observed in BTX-A 300U versus 200U (MD = 84.13, 95% CI: 35.74, 132.51) (**Table 3**).

#### Detrusor Compliance (DC)

##### DC for NDO in Short-Term Observation Period

At 6 weeks. Two included studies (Cruz et al., 2011; Rovner et al., 2013) reported this outcomes. **Table 3** showed that BTX-A groups (200U and 300U) significantly increased DC compared to placebo (200U: MD = 66.29, 95% CI: 44.24, 88.34; 300U: MD = 56.51, 95% CI: 35.00, 78.03). However, no significant difference was obtained in different dosages of BTX-A (300U vs. 200U: MD = -9.79, 95% CI: -35.00, 15.42) (**Table 3**).

##### DC for IOAB in Short-Term Observation Period

At 12 weeks. There was only one study (Rovner et al., 2011) reported this outcome. All of the subgroups showed no significant difference (BTX-A 50U vs. Placebo, MD = 65.50, 95% CI: 23.24, 107.76; 100U vs. Placebo, MD = 85.80, 95% CI: 41.31, 130.29; 200U vs. Placebo, MD:104.50, 95% CI: 47.52, 161.48; 300U vs. Placebo, MD = 75.80, 95% CI: 23.42, 128.18; 200U vs. 150U, MD = 68.10, 95% CI: 5.24, 130.96; 150U vs. Placebo, MD:36.40, 95% CI: -8.85, 81.65) (**Table 4**).

##### DC for IOAB in Long-Term Observation Period

At 36 weeks. There was only one included study (Rovner et al., 2011) reported this outcome. Statistically significant difference was observed in three subgroups (BTX-A 50U vs. Placebo, MD = 60.30, 95% CI: 16.08, 104.52; BTX-A 200U vs. Placebo, MD = 64.80, 95% CI: 4.24, 125.36; BTX-A 300U vs. Placebo, MD = 60.80, 95% CI: 11.64, 109.96). However, no significant difference was observed in other subgroups (**Table 4**).

### Safety

All of the effects on adverse events in NDO and IOAB patients were shown in **Table 3** and **Table 4**, respectively.

#### Urinary Tract Infections (UTI)

##### UTI for NDO

Seven included studies (Schurch et al., 2005; Cruz et al., 2011; Herschorn et al., 2011; Ginsberg et al., 2012; Ginsberg et al., 2013; Rovner et al., 2013; Chen & Kuo, 2014) reported the UTI events in NDO patients. And significant effect on increasing UTI was observed in BTX-A 200U (RR = 1.44, 95% CI: 1.27, 1.62) and 300U (RR = 1.51, 95% CI: 1.35, 1.70), compared to placebo. Nevertheless, no significant effect was obtained between BTX-A 300U and 200U (RR = 1.07, 95% CI: 0.97, 1.18) (**Table 3**).

##### UTI for IOAB

Eight included studies (Brubaker et al., 2008; Dmochowski et al., 2010; Altaweel et al., 2011; Denys et al., 2012; Chapple et al., 2013; Nitti et al., 2013; Sievert et al., 2014; Abdelwahab et al., 2015) reported this outcome. Four of 15 subgroups showed a significant effect on increasing UTI, compared to placebo (BTX-A 100U: RR = 2.55, 95% CI: 2.09, 3.12; 150U: RR = 2.36, 95% CI: 1.19, 4.68; 200U: RR = 2.68, 95% CI: 1.46, 4.93; 300U: RR = 2.12, 95% CI: 0.98, 4.58). And no significant difference was observed in the remain 11 subgroups (**Table 4**).

#### Urinary Retention

##### Urinary Retention of NDO

Four included studies (Cruz et al., 2011; Ginsberg et al., 2012; Ginsberg et al., 2013; Rovner et al., 2013) reported available data of this outcome. Significant effect of BTX-A at 200U and 300U was observed compared to placebo (RR = 5.85, 95% CI: 3.84, 8.91; RR = 6.78, 95% CI: 4.46, 10.30). Nevertheless, the effect between BTX-A 300U and 200U didn’t reach significance (RR = 1.16, 95% CI: 0.95, 1.43) (**Table 3**).

##### Urinary Retention of IOAB

Four included studies (Dmochowski et al., 2010; Chapple et al., 2013; Nitti et al., 2013; Sievert et al., 2014) reported this outcome. And in 6 of 15 subgroups, BTX-A demonstrated a significant effect on increasing urinary retention (BTX-A 100U vs. Placebo: RR = 13.99, 95% CI: 5.71, 34.30; 150U vs. Placebo: RR = 12.04, 95% CI: 1.65, 87.85; 200U vs. Placebo: RR = 9.92, 95% CI: 1.34, 73.29; 300U vs. Placebo: RR = 10.95, 95% CI: 1.50, 80.00; 150U vs. 50U: RR = 3.14, 95% CI: 1.22, 8.09; 300U vs. 50U: RR = 2.85, 95% CI: 1.10, 7.38) (**Table 4**).

#### Hematuria

##### Hematuria of NDO

Seven included studies (Schurch et al., 2005; Cruz et al., 2011; Herschorn et al., 2011; Ginsberg et al., 2012; Ginsberg et al., 2013; Rovner et al., 2013; Chen & Kuo, 2014) reported this outcome. The significant difference was only observed in BTX-A 300U, compared to placebo (RR = 1.97, 95% CI: 1.24, 3.13). For the others, no significant difference was observed (BTX-A 200U vs. Placebo: RR = 1.59, 95% CI: 0.97, 2.62 and 300U vs. 200U: RR = 1.22, 95% CI: 0.83, 1.80) (**Table 3**).

##### Hematuria of IOAB

Two included studies (Chapple et al., 2013; Sievert et al., 2014) reported this outcome. Nevertheless, no significant effect was observed in BTX-A 100U compared to placebo (RR = 1.38, 95% CI: 0.78, 2.42) (**Table 4**).

#### PVR-Related Catheterization of IOAB

Five included studies (Sahai et al., 2007; Brubaker et al., 2008; Cohen et al., 2009; Dmochowski et al., 2010; Denys et al., 2012; Nitti et al., 2013) reported the available data for PVR-related catheterization in IOAB patients. Significant effect was observed in 2 of 15 subgroups (BTX-A 200U vs. Placebo: RR = 15.74, 95% CI: 3.13, 79.31; BTX-A 200U vs. 50U: RR = 3.95, 95% CI: 1.17, 13.37). And the effect of remain 13 subgroups didn’t reach significance (**Table 4**).

#### Muscle Weakness of NDO

Four included studies (Cruz et al., 2011; Herschorn et al., 2011; Ginsberg et al., 2012; Rovner et al., 2013) reported the available data for muscle weakness in NDO patients. Significant effect on increasing muscle weakness in BTX-A at 300U was observed compared to placebo (RR = 3.01, 95% CI: 1.50, 6.02), however, the effect of BTX-A at 200U was not significant (RR = 1.53, 95% CI: 0.76, 3.06). Furthermore, the effect of 300U was statistic significant compared to 200U (RR = 1.75, 95% CI: 1.03, 2.97) (**Table 3**).

## Discussion

Based on the American Urological Association (AUA) guidelines (Gormley et al., 2015) for management of OAB patients, the standard treatment, including education, behavior therapies, pharmacotherapy, and BTX-A, has been widely used for OAB (Engeler et al., 2015; Zhou et al., 2015; Krhut et al., 2016). We performed this meta-analysis with the most up-to-date and comprehensive evidence for evaluating clinical benefit of BTX-A in UI in patients with NDO and IOAB. In order to reflect the effects more accurately, the different follow-up periods and dosages were analyzed. Ultimately, we found that the BTX-A demonstrated a significant effect on reducing episodes of UI and MDP in NDO patients, and BTX-A increased DC in IOAB patients, however, the adverse events in two groups increased in this meta-analysis.

Five kinds of dosages of BTX-A for NDO and IOAB (50U, 100U, 150U, 200U, and 300U) and placebo form 19 included studies considered as high quality under quality assessment were analyzed for evaluating the effectiveness. The results demonstrated that BTX-A 300U and 200U is superior to placebo in the protective role against UI episodes per week in NDO patients during follow-up period (2, 6, and 12 weeks), which was considered as a satisfactory outcome and enable BTX-A to be considered as intervention for management of UI. Furthermore, BTX-A reduced MDP during 6 weeks follow-up compared to placebo, which might account for the increase in PVR in both 200U and 300U, and the 300U was worse than 200U. The DC in NDO was increased at both BTX-A 200U and 300U, which might account for the reduction in episodes of UI. These results were consistent with other similar meta-analyses (Zhou et al., 2015; Cheng et al., 2016). However, the BTX-A 300U showed no superior effect on UI, MDP, and DC compared to 200U, with increased UTI, urinary retention, muscle weakness, and hematuria, thus, the BTX-A 300U was not recommended in management of UI in NDO patients in this study.

Furthermore, the results also revealed that dosages of BTX-A not less than 50U were superior to placebo in the improvement of DC in IOAB at 12 and 36 weeks, except the BTX-A 150U and 100U at 36 weeks and BTX-A 150U at 12 weeks in this study. This superior effect was consistent with the Rovner’s study (Rovner et al., 2011) which has reported that BTX-A at dosages of more than 100U showed significant improvement in OAB. It was also observed between BTX-A 200U and 300U for UI episodes per week at 2 weeks in NDO patients, which may be caused by small sample size. However, compared with placebo, five dosages of BTX-A demonstrated no significant difference in for MDP in IOAB at 12 and 36 weeks, which was not consistent with Chapple’s study (Chapple et al., 2013) reported OAB patients could be benefit from BTX-A 100U. In addition, BTX-A 200U and 300U had nearly same effectiveness on NDO and IOAB. Although Nuanthaisong’s study (Nuanthaisong et al., 2014) reported that BTX-A over 360U was an effective treatment, we mainly focused on dosages not more than 300, especially 300 and 200U for NDO and IOAB, thus, the studies exploring effect of dosages of BTX-A more than 300U are required in further research. All these results suggested that BTX-A 300U and 200U is superior to placebo in effectiveness on NDO at short-term observations, especially at 6 weeks. Remarkably, in consideration that UI was regarded as a chronic disease, and BTX-A 200U also contributed to increase DC in IOAB patients during 36 weeks, therefore, the researches exploring long-term effect of BTX-A are required in future.

Another essential part of this study was the assessment of safety of BTX-A, and we found that basically all the adverse events were increased in both NDO and IOAB patients in this meta-analysis. The results showed that possibility of suffering UTI was higher in both BTX-A 300U and 200U in NDO than placebo, but the BTX-A 300U was not higher than 200U. This effect might due to the markedly increased PVR and urinary retention. Therefore, the NDO patients treated with BTX-A must be aware of the potential risk of UTI, and the prophylactic antibiotics could be considered. These complications could account for the increased risk of hematuria as consequence, except in BTX-A 200U. And no effect on increasing risk of muscle weakness was observed in BTX-A 200U in NDO patients, however the BTX-A 300U significantly increased this risk compared to both placebo and BTX-A 200U. For IOAB patients, almost every dosages of BTX-A, except at 50U, demonstrated a higher risk of urinary retention compared to placebo. Furthermore, the increased dosages basically did not increase the risk of urinary retention. Therefore, in theory, the different dosages would not increase the risk of UTI as consequence, which was consistent with the results of this study that the risk of suffering UTI was higher than placebo, however, no increased risk was observed in different dosages. In addition, no requirement of PVR-related catheterizations was observed in IOAB patients compared to placebo, except at dosage of 200U. The exception and heterogeneity may be caused by small sample size. Although BTX-A was superior to placebo, indicating that BTX-A had slightly more adverse events than placebo. However, the localized urologic events, which considered as main adverse effects of BTX-A, were not found. It might due to the well-tolerated, however, more researches are still required in this field.

The advantages in this study are as follows. Firstly, Chinese articles were also reviewed in this meta-analysis as well as English studies, unfortunately, there were no studies met the inclusion criteria. Secondly, BTX-A at different dosages were investigated, and the clinical benefit of BTX-A was assessed based on comprehensive measurement of outcomes. Thus, the most up-to-date evidence has been provided for clinical practice and medical guidelines. Meanwhile, different dosages were evaluated to assess the safety. In our study, the results demonstrated that the increase of dosage of BTX-A has no protective effect of complications. For effectiveness, although the BTX-A has a protective effect for NDO, different dosage showed no significant discrepancy in UI episodes, MDP and DC, except PVR. For IOAB, the most remarkable outcome is the difference of PVR for injecting diverse dosage. In order to detail the long-term potential impact, we comprehensively and systematically evaluated BTX-A in treatment of UI in patients with NDO and IOAB for short- and long-term observation periods, which could reduce the risk of from correct outcomes as well. Although significant differences were not obtained in our study, we offered a potential possibility for other researcher to investigate this field for the prognosis of patients. Furthermore, results of subgroup analyses based on dosages and types of OAB objectively disclosure true the clinical benefit, and it has guiding clinicians to treat NDO and IOAB. After all, Surgery may be carried out as a last resort (Krhut et al., 2016), resulting in trauma, thus the drug treatment of OAB requires urgent attention.

There are also several limitations in this study. Firstly, insufficient sample size and RCTs is the main disadvantages in our study, caused by the study design of original studies. For example, we excluded one study reported results 72 h after injection, which is possible to ignore the long-term impact which might produce the result of greater difference. Due to the limitations of language, the studies using other languages (except English and Chinese) were ignored. Secondly, benign prostatic hyperplasia (BPH) is a prominent factor, resulting OAB. The pathogenesis was failed to be executed using subgroup analyses, because of the lack of relevant data. The data of short-term and long-term observations periods were insufficient in assess the clinical benefits of BTX-A. Therefore, more high-quality larger RCTs with short-term and long-term observations periods should be added to assess of the clinical benefits of BTX-A from different perspectives.

## Conclusion

This meta-analysis indicates that BTX-A 300U and 200U showed a positive effect on management of UI NDO for short-term therapy based on current evidence. In addition, clinical benefits were not found in BTX-A 50U, 100U, 150U, 200U, and 300U for IOAB for long-term treatment, except the efficacy of BTX-A for DC. There is a significant finding that dosages 300U and 200U could improve DC of IOAB for short- and long-term treatments, compared to placebo. Furthermore, BTX-A 300U and 200U have no significant difference in adverse events. Therefore, we recommend that BTX-A 200U could be considered as intervention for UI in patients with NDO for the short-term treatments, with minimal, local, and manageable adverse events. The long-term treatments of BTX-A for UI in NDO patients and short-term treatments for IOAB patients require more RCTs to be investigated.

## Author Contributions

Q-QG, X-YD, Y-QX and JX had full access to the data and take responsibility for the integrity and accuracy of the data analysis. Q-QG, X-YD and JX were responsible for the study concept and design. CG, Y-QX and JX investigators/collaborators listed below were involved in the acquisition of data. All the authors contributed to the analysis and interpretation of data and to the critical revision of manuscript. CG drafted the manuscript.

## Conflict of Interest

The authors declare that the research was conducted in the absence of any commercial or financial relationships that could be construed as a potential conflict of interest.
