# Supplementary material for: Meta-Analysis of Randomized Controlled Trials Using Botulinum Toxin A at Different Dosages for Urinary Incontinence in Patients With Overactive Bladder
Source: Front Pharmacol. 2020 Jan 15;10:1618. doi: 10.3389/fphar.2019.01618 (PMC6974921; doi:10.3389/fphar.2019.01618)
Supplement: Supplementary file 1 [file DataSheet_1.docx]

**Supplemental File**

**Supplement Method 1** Search strategies

**Supplement Method 2** Reason from exclusion of literature

**Supplement Method 1** Search strategies

**1. Ovid MEDLINE(R) and Epub Ahead of Print, In-Process & Other Non-Indexed Citations, Daily, and Versions(R) <1946 to July 31, 2018>**

1. Urinary bladder.mp.
2. Urinary bladder.ti,ab.
3. Bladder, Urinary.ti,ab.
4. Overactive bladder.mp.
5. Urinary Bladder, Overactive.ti,ab.
6. Overactive Urinary Bladder.ti,ab.
7. Bladder, Overactive.ti,ab.
8. Overactive Detrusor.ti,ab.
9. Neurogenic detrusor overactivity.ti,ab.
10. Overactive Detrusor Function.ti,ab.
11. Detrusor Function, Overactive.ti,ab.
12. Idiopathic overactive bladder.ti,ab.
13. Overactive detrusor.mp.
14. Overactive detrusor.ti,ab.
15. Urinary incontinence.mp.
16. #1 or #2 or #3 or #4 or #5 or #6 or #7 or #8 or #9 or #10 or #11 or #12 or #13 or #14 or #15
17. Botulinum Toxins, Type A.mp.
18. Botulinum Neurotoxin A.ti,ab.
19. Neurotoxin A, Botulinum.ti,ab.
20. Clostridium botulinum A Toxin.ti,ab.
21. Clostridium Botulinum Toxin Type A.ti,ab.
22. Botulinum A Toxin.ti,ab.
23. Toxin, Botulinum A.ti,ab.
24. Botulinum Toxin Type A.ti,ab.
25. Meditoxin.ti,ab.
26. Neuronox.ti,ab.
27. Oculinum.ti,ab.
28. Clostridium botulinum type A.mp.
29. Clostridium botulinum A.ti,ab.
30. #17 or #18 or #19 or #20 or #21 or #22 or #23 or #24 or #25 or #26 or #27 or #28 or #29
31. #16 and #30
32. limit 31 to (("young adult (19 to 24 years)" or "adult (19 to 44 years)" or "young adult and adult (19-24 and 19-44)" or "middle age (45 to 64 years)" or "middle aged (45 plus years)" or "all aged (65 and over)" or "aged (80 and over)") and english and humans and randomized controlled trial)

**2. EMbase + EMbase classic <1946 to July 31, 2018>**

1. 'Urinary bladder'/exp
2. 'Urinary bladder':ti,ab
3. 'Bladder, Urinary':ti,ab
4. 'Overactive bladder'/exp
5. 'Urinary Bladder, Overactive':ti,ab
6. 'Overactive Urinary Bladder':ti,ab
7. 'Bladder, Overactive':ti,ab
8. 'Overactive Detrusor':ti,ab
9. 'Neurogenic detrusor overactivity':ti,ab
10. 'Overactive Detrusor Function':ti,ab
11. 'Detrusor Function, Overactive':ti,ab
12. 'Diopathic overactive bladder':ti,ab
13. 'Overactive detrusor'/exp
14. 'Overactive detrusor':ti,ab
15. 'Urinary incontinence'/exp
16. #1 OR #2 OR #3 OR #4 OR #5 OR #6 OR #7 OR #8 OR #9 OR #10 OR #11 OR #12 OR #13 OR #14 OR #15
17. 'Botulinum Toxins, Type A'/exp
18. 'Botulinum Neurotoxin A':ti,ab
19. 'Neurotoxin A, Botulinum':ti,ab
20. 'Clostridium botulinum A Toxin':ti,ab
21. 'Clostridium Botulinum Toxin Type A':ti,ab
22. 'Botulinum A Toxin':ti,ab
23. 'Toxin, Botulinum A':ti,ab
24. 'Botulinum Toxin Type A':ti,ab
25. 'Meditoxin':ti,ab
26. 'Neuronox':ti,ab
27. 'Oculinum':ti,ab
28. 'Clostridium botulinum type A'/exp
29. 'Clostridium botulinum A':ti,ab
30. #17 OR #18 OR #19 OR #20 OR #21 OR #22 OR #23 OR #24 OR #25 OR #26 OR #27 OR #28 OR #29
31. #16 AND #30
32. #31 AND [randomized controlled trial]/lim AND [english]/lim AND ([young adult]/lim OR [adult]/lim OR [middle aged]/lim OR [aged]/lim OR [very elderly]/lim) AND [humans]/lim AND ([embase]/lim OR [embase classic]/lim)

**3. CENTRAL, The** **Cochrane Library <Issue 7 of 12, June 2018>**

1. MeSH descriptor: [Urinary bladder] explode all trees
2. MeSH descriptor: [Overactive bladder] explode all trees
3. MeSH descriptor: [Overactive detrusor] explode all trees
4. MeSH descriptor: [Urinary incontinence] explode all trees
5. (Urinary bladder):ti,ab,kw
6. (Bladder, Urinary):ti,ab,kw
7. (Urinary Bladder, Overactive):ti,ab,kw
8. (Overactive Urinary Bladder):ti,ab,kw
9. (Bladder, Overactive):ti,ab,kw
10. (Overactive Detrusor):ti,ab,kw
11. (Neurogenic detrusor overactivity):ti,ab,kw
12. (Overactive Detrusor Function):ti,ab,kw
13. (Detrusor Function, Overactive):ti,ab,kw
14. (Idiopathic overactive bladder):ti,ab,kw
15. (Overactive detrusor):ti,ab,kw
16. #1 or #2 or #3 or #4 or #5 or #6 or #7 or #8 or #9 or #10 or #11 or #12 or #13 or #14 or #15
17. MeSH descriptor: [Botulinum Toxins, Type A] explode all trees
18. (Botulinum Neurotoxin A):ti,ab,kw
19. (Neurotoxin A, Botulinum):ti,ab,kw
20. (Clostridium botulinum A Toxin):ti,ab,kw
21. (Clostridium Botulinum Toxin Type A):ti,ab,kw
22. (Botulinum A Toxin):ti,ab,kw
23. (Toxin, Botulinum A):ti,ab,kw
24. (Botulinum Toxin Type A):ti,ab,kw
25. (Meditoxin):ti,ab,kw
26. (Neuronox):ti,ab,kw
27. (Oculinum):ti,ab,kw
28. MeSH descriptor: [Clostridium botulinum type A] explode all trees
29. (Clostridium botulinum A):ti,ab,kw
30. #17 or #18 or #19 or #20 or #21 or #22 or #23 or #24 or #25 or #26 or #27 or #28 or #29
31. #16 and #30

**4. China National Knowledge Internet, <Issue 7 of 12, June 2018>**

1. 尿失禁
2. 膀胱过度活动综合征
3. 神经性尿失禁
4. 特异性尿失禁
5. #1 OR #2 OR #3 OR #4
6. 肉毒素
7. 肉毒杆菌内毒素
8. 肉毒杆菌素
9. #6 OR #7 OR #8
10. #5 AND #9

**5. WanFang database, <Issue 7 of 12, June 2018>**

1. 尿失禁
2. 膀胱过度活动综合征
3. 神经性尿失禁
4. 特异性尿失禁
5. #1 OR #2 OR #3 OR #4
6. 肉毒素
7. 肉毒杆菌内毒素
8. 肉毒杆菌素
9. #6 OR #7 OR #8
10. #5 AND #9

**Supplement Method 2** Reason from exclusion of literature

| **Study** | **Year** | **Title** | **Reasons** |
| --- | --- | --- | --- |
| Komesu,Y | 2018 | Refractoryurgency urinary incontinence treatment in women: impact of age on outcomes and complications | The population wasn't eligible. |
| Liu Jing | 2018 | (Chinese) Curative effect observation of botulinum toxin detrusor injection in neurogenic detrusor overactivity | The deviation was unable to be calculated. |
| ZHONG Haihu | 2018 | (Chinese) Comparison of the Efficacy of Detrusor Injection of BTX-A in the Treatment of IDO and NDO | The study design wasn't meet the inclusion criteria. |
| Kennelly,M | 2017 | Efficacy and safety of onabotulinumtoxinA therapy are sustained over 4 years of treatment in patients with neurogenic detrusor overactivity: final results of a long-term extension study | The study design wasn't meet the inclusion criteria. |
| Herschorn,S | 2017 | The Efficacy and Safety of OnabotulinumtoxinA or Solifenacin Compared with Placebo in Solifenacin Naïve Patients with Refractory Overactive Bladder: Results from a Multicenter, Randomized, Double-Blind Phase 3b Trial | The population wasn't eligible. |
| Denys,P | 2016 | Positive outcomes with first onabotulinumtoxinA treatment persist in the long term with repeat treatments in patients with neurogenic detrusor overactivity | The intervention wasn't met the inclusion criteria. |
| Visco,A | 2016 | Cost-Effectiveness Analysis of Anticholinergics Versus Botox for Urgency Urinary Incontinence: Results From the Anticholinergic Versus Botox Comparison Randomized Trial | The study design wasn't meet the inclusion criteria. |
| Martins,S | 2016 | Efficacy and Safety of OnabotulinumtoxinA in Patients with Urinary Incontinence Due to Neurogenic Detrusor Overactivity: update of the Pivotal Randomised, Double-blind, Placebo-controlled Trials | The study design was updata abstract. |
| TANG Cai | 2016 | (Cinese) Efficacy of diffreent dose of Botulinum toxin A in treatment of patients with neurogenic detrusor overactivity | The study design wasn't meet the inclusion criteria. |
| Krhut,J | 2015 | Intravesical instillation of onabotulinum toxin A embedded in inert hydrogel in the treatment of idiopathic overactive bladder: A double-blind randomized pilot study | The study design wasn't meet the inclusion criteria. |
| Fu | 2015 | Efficacy of Botulinum-A toxin injection into bladder to treat neurogenic incontinence in patients with spinal cord injury: comparison of two doses | The study design wasn't meet the inclusion criteria. |
| Fu Guang | 2015 | (Chinese)Efficacy of Botulinum-A toxin injection into bladder to treat neurogenic incontinence in pafients with spinal cord injury: comparison of two doses | The deviation was unable to be calculated. |
| XieKeji | 2015 | (Chinese) Effect and safety of trigone-including intradetrusor injection of botulinum toxin A in patients with eurogenic detrusor overactivity and incontinence: a prospective, multicenter, single-blind and randomized controlled trial | The study design wasn't meet the inclusion criteria. |
| Kuo,H | 2014 | Pilot study of liposome-encapsulated onabotulinumtoxina for patients with overactive bladder: a single-center study | The study design wasn't meet the inclusion criteria. |
| Samal,V | 2013 | Submucosal administration of onabotulinumtoxinA in the treatment of neurogenic detrusor overactivity: pilot single-centre experience and comparison with standard injection into the detrusor | The study design wasn't meet the inclusion criteria. |
| Nitti,V | 2013 | Clinically meaningful improvements in urinary incontinence and quality of life over multiple treatment cycles of onabotulinumtoxina in patients with overactive bladder syndrome and urinary incontinence: interim analysis of a long-term extension study | The study design wasn't meet the inclusion criteria. |
| Chartier,K | 2013 | Sustained improvements in health-related quality of life following repeat onabotulinumtoxina detrusor injections in patients with neurogenic detrusor overactivity: results from two phase 3 studies | The study design wasn't meet the inclusion criteria. |
| Tincello,D | 2012 | Botulinum toxin a versus placebo for refractory detrusor overactivity in women: a randomised blinded placebo-controlled trial of 240 women | The population wasn't eligible. |
| Kanagarajah, P | 2012 | Role of botulinum toxin-A in refractory idiopathic overactive bladder patients without detrusor overactivity | The study design wasn't meet the inclusion criteria. |
| Granese, R | 2012 | Botox for idiopathic overactive bladder: efficacy, duration and safety. Effectiveness of subsequent injection | The study design wasn't meet the inclusion criteria. |
| Dowson, C | 2011 | The safety and efficacy of botulinum toxin-A in the management of bladder oversensitivity: a randomised double-blind placebo-controlled trial | The population wasn't eligible. |
| Safari, S | 2010 | Intravesical injections of botulinum toxin type A for management of neuropathic bladder: a comparison of two methods | The study design wasn't meet the inclusion criteria. |
| Grise, Philippe | 2010 | Efficacy and tolerability of botulinum toxin type A in patients with neurogenic detrusor overactivity and without concomitant anticholinergic therapy: comparison of two doses | The intervention wasn't met the inclusion criteria. |
| Gousse, A | 2010 | A single center, prospective, randomized study to evaluate the effect of repeat intradetrusor injections of botulinum toxin-A for refractory idiopathic overactive bladder patients: dose difference between 100 U vs 150 U (Abstract number 65) | The study design was abstract. |
| Flynn, M | 2009 | Outcome of a randomized, double-blind, placebo controlled trial of botulinum A toxin for refractory overactive bladder | The study design wasn't meet the inclusion criteria. |
| Ehren, I | 2007 | Efficacy and impact of botulinum toxin A on quality of life in patients with neurogenic detrusor overactivity: A randomized, placebo-controlled, double-blind study | The intervention wasn't met the inclusion criteria. |
| Li Xin-de | 2007 | (Chinese) Intravesical injections of botulinum-A toxin for treating female overactive bladder | The study design wasn't meet the inclusion criteria. |
| Finney, S | 2006 | A double-blind, placebo controlled study investigating efficacy of botulinum toxin type A (Dysport - trademark) in MS related overactive bladder sydrome (OAB): provisional 36 week results | The study design wasn't meet the inclusion criteria. |
| ZUO Yi-gang | 2006 | (Chinese) Urethral injection of botulinum toxin A in the treatment of urge urinary incontinence due to detrusor hyperreflexia | The study design wasn't meet the inclusion criteria. |
| ZUO Yi-gang | 2005 | (Chinese) Treatment of urge urinary incontinence caused by detrusor hyperreflexia with injecting botulinum toxin A into the detrusor under the guide of cystoscopy | The study design wasn't meet the inclusion criteria. |
| LIAO Limin | 2005 | (Chinese) Treatment of neurogenic bladder associated with spinal cord injury wmI botulinum-A toxin injected into bladder wall | The study design wasn't meet the inclusion criteria. |
| Giannantoni, A | 2004 | New therapeutic options for refractory neurogenic detrusor overactivity | The study design wasn't meet the inclusion criteria. |
| Giannantoni, A | 2004 | Intravesical resiniferatoxin versus botulinum-A toxin injections for neurogenic detrusor overactivity: a prospective randomized study | The study design wasn't meet the inclusion criteria. |
| Gallien | 1998 | Treatment of detrusor sphincter dyssynergia by transperineal injection of botulinum toxin | The study design wasn't meet the inclusion criteria. |
